# Preoperative C-Reactive Protein as a Risk Factor for Postoperative Delirium in Elderly Patients Undergoing Laparoscopic Surgery for Colon Carcinoma

**DOI:** 10.1155/2017/5635640

**Published:** 2017-10-18

**Authors:** Dong Xiang, Hailin Xing, Huiyu Tai, Guozhu Xie

**Affiliations:** ^1^Department of Anesthesiology, Taizhou People's Hospital, Medical School of Nantong University, Taizhou, China; ^2^Department of Intensive Care Unit, Taizhou People's Hospital, Medical School of Nantong University, Taizhou, China

## Abstract

**Background:**

Postoperative delirium (POD) is a very common complication in operative disciplines, especially in those elderly patients after cardiac surgery. This study aimed to investigate the relationship between C-reactive protein (CRP) and POD in elderly patients undergoing laparoscopic surgery for colon carcinoma.

**Methods:**

160 elderly patients scheduled to undergo selective laparoscopic surgery for colon carcinoma were prospectively recruited in this present study. The preoperative demographic and medical characteristics, intraoperative variables, and postoperative complications were all recorded in detail. POD assessment was performed once a day for the first 3 days and at 7th day after surgery, respectively. CRP concentrations preoperatively and on postoperative days 1, 2, and 3 were measured by using human enzyme linked immunosorbent assay (ELISA).

**Results:**

Of all the 160 enrolled patients, 39 had suffered POD with a POD incidence of 24.4% within the first week after the operation. The univariate analysis and multiple logistic regression analysis suggested preoperative CRP concentrations as the only independent predicator for POD in patients undergoing laparoscopic surgery for colon carcinoma (OR: 5.87; 95% CI: 2.22–11.4; *P* = 0.018).

**Conclusions:**

This present study highlighted the predictive role of preoperative CRP concentrations for POD in elderly patients undergoing laparoscopic surgery for colon carcinoma.

## 1. Introduction

Postoperative delirium (POD) is a very common complication in operative disciplines, especially in those elderly patients after cardiac surgery [1]. The described prevalence of POD varies between 30 and 80% in elderly patients after cardiac surgeries [2, 3] and 15%–53% in elderly surgical patients [4]. Numerous studies have revealed that POD is significantly associated with increased complication incidence, long-term cognitive impairment, prolonged hospital length of stay, elevated costs, and overall mortality [5–7]. To predict the implications of POD and improve the quality of care, attempting to determine independent risk factors for POD is of great importance. A number of previous studies have been performed regarding predicative factors for POD; however no consensus has been made until now probably due to the complicated pathogenesis of POD [8]. Previous studies have reported that delirium is associated with elevated proinflammatory cytokines [9] and proteins involved in the stress response [10] in medical or surgical patients. C-reactive protein (CRP), one of the most common markers for systemic inflammation, has been indicated as independent risk factor for delirium following vascular surgery [11] and hip surgery [12]. However, the relationship between CRP and POD in patients undergoing laparoscopic surgery for colon carcinoma still remains relatively unknown, which was just the objective of this present study.

## 2. Material and Methods

### 2.1. Patients

**Figure 1:**
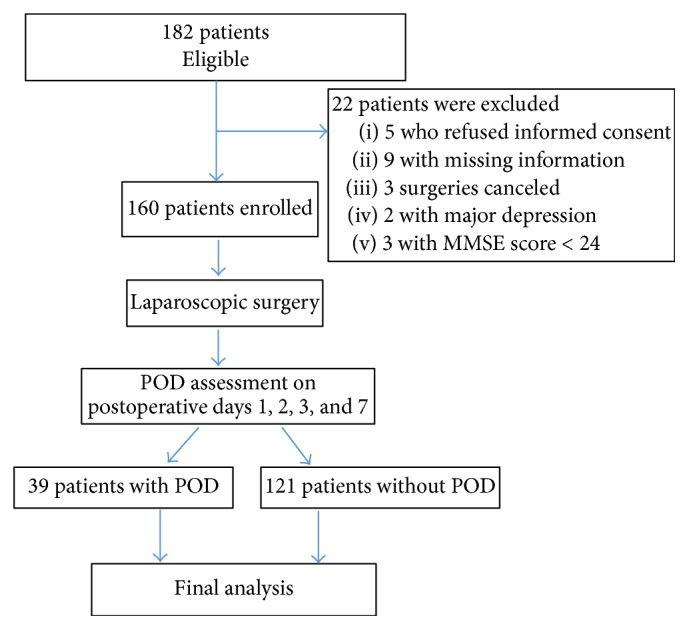
Patient CONSORT. MMSE, Mini-Mental State Examination; POD, postoperative delirium.

This present study protocol was approved by the Medical Institutional Ethics Committee of Jiangsu province and Taizhou People's Hospital. Those elderly patients (aged ≥ 65 years) scheduled to undergo selective laparoscopic surgery for colon carcinoma in Taizhou People's Hospital from April, 2014, to January, 2017, were prospectively recruited in this present study. All the participants were required to offer the signed informed consent. Exclusion criteria were described as follows: (1) with major depression; (2) with preexisting or a history of dementia delirium; (3) with cognitive impairment which was defined with a Modified Mini-Mental State Examination (MMSE) score < 24; (4) with clinically neurologic disorder or psychosis. 182 eligible patients were included into our study; 22 of them were excluded for varied reasons (informed consent refusal, missing information, etc.). In total, 160 elderly patients undergoing laparoscopic surgery for colon carcinoma were included into the final analysis, which was shown in the patient CONSORT (Figure 1).

### 2.2. Methods

Demographic and medical characteristics (including age, gender, and education) were evaluated. The modified Charlson's Comorbidity Index (MCCI) was utilized for the medical comorbidities assessment by summing points [13]. POD was evaluated using the Confusion Assessment Method-Intensive Care Unit (CAM-ICU) by calculating CAM scores [14]. POD assessment was performed once a day (in the evening) for the first 3 days and at 7th day after surgery, respectively. A positive POD diagnosis was given when patients had a positive result at least for once within 1 week of the assessment. The intraoperative variables (operation time, anesthesia time, blood loss, etc.), postoperative complications (wound infection, urinary tract infection, pulmonary infection, etc.), and postoperative adverse cardiovascular events (such as myocardial infarction, arrhythmias, and heart failure) were also detailed, recorded, and analyzed.

To avoid the interferential impacts by anesthesia, all the enrolled patients underwent the operation under general anesthesia by the same anesthesia team. With no premedication, intravenous midazolam, propofol, sufentanil, and rocuronium were used for inducing anesthesia. Anesthesia was maintained with sevoflurane, propofol, remifentanil, and dexmedetomidine. The serial blood collection preoperatively and on postoperative days 1, 2, and 3 was conducted from all enrolled participants. Blood samples were stored on ice in heparinized tubes and immediately centrifuged (1500*g* at 4°C for 15 minutes). The separated plasma samples from cellular material were then stored at −80°C until assayed. CRP concentrations were measured by using human enzyme linked immunosorbent assay (ELISA) kit (R&D Systems, Minneapolis, MN, USA). The ELISA was carried out in accordance with the manufacturers' instructions by the same laboratory assistant who was completely blinded to this study.

### 2.3. Statistical Analysis

The data analysis was performed using SPSS 19.0 (SPSS Inc., Chicago, IL, USA). Categorical data were expressed as number (with percentage, *n*%) and compared with Chi-square test or Fisher exact test. Continuous data were presented as mean levels (with standard deviation) or median (with interquartile range) and compared via the Mann–Whitney *U*-test or Student's *t*-test appropriately. The univariate and multiple logistic regression analyses were plotted to evaluate the predicative validity of pre-, intra-, or postoperative variables for POD. All statistical tests were bilateral probability and *P* < 0.05 was considered significant.

## 3. Results

### 3.1. Preoperative Variables

The demographic and clinical characteristics of the enrolled elderly patients were exhibited in Table 1 in detail. 39 of the 160 patients had suffered POD with a POD incidence of 24.4%, which was similar to other previous studies [15]. The mean age of all the enrolled patients was 70.1 years, and significant difference in age between the patients with or without POD was found. The preoperative MCCI score was significantly higher in patients who suffered POD when compared with those without POD. In addition, the preoperative MMSE score and percentage of heavy drinkers were significantly higher in the delirious group than the nondelirious group. No statistically significant differences were found in the gender, education, body mass index, smoking habits, and ASA physical status between the patients with POD or not.

### 3.2. Intraoperative Variables

When compared with patients without POD, those with POD showed significantly longer operation time and anesthesia time. As shown in Table 1, the operative approach was also associated with the incidence of POD.

### 3.3. Postoperative Complications

A significant difference exists between the patients in delirious group or nondelirious group with respect to postoperative incision infection. The incidence of pulmonary infection or cardiovascular events after the surgery seemed significantly associated with the occurrence of POD. When we compared other postoperative complications, no significant differences were observed between groups with respect to the prevalence of urinary tract infection, bowel obstruction, anastomotic leakage, and postoperative bleeding.

### 3.4. Plasma CRP Concentrations and POD

The postoperative CRP concentrations were many times as high as preoperative levels, which indicated a strong effect of operation on CRP concentrations. The results also revealed that patients with POD showed a significantly higher CRP concentration preoperatively and on postoperative day 2 than those without POD.

### 3.5. Logistic Regression Analysis for POD

As shown in Table 2, all potential predicative factors mentioned above were summarized by univariate analysis. The results from the univariate logistic regression analysis indicated that age, MCCI, preoperative MMSE score, postoperative cardiovascular events, and CRP concentrations preoperatively or on postoperative day 2 were associated with POD. With these six variables introduced into the final multivariate analysis, the results suggested preoperative CRP concentrations as the independent predicator for POD in patients undergoing laparoscopic surgery for colon carcinoma (OR: 5.87; 95% CI: 2.22–11.4; *P* = 0.018).

## 4. Discussion

To our knowledge, this current study demonstrated that plasma CRP concentrations emerged as an independent factor for POD in the elderly patients undergoing laparoscopic surgery for colon carcinoma for the first time. Recently published studies have indicated a positive correlation between POD and early mortality after surgery, which emphasizes the importance of POD prediction.

Previous studies have revealed age as a well-established predictor for POD with no certain mechanisms [16, 17]. In this present study, the patients who underwent POD also had a higher age than those without POD. However, the final multivariate analysis did not indicate age as a predicator as expected, which might be explained by the small age range or different operation types of the enrolled participants. Preoperative cognitive function decline is considered as a risk factor for postoperative cognitive problems and later life equality [18]. Our results from univariate analysis instead of multivariate analysis also showed a close association between preoperative MMSE score and POD. Previous studies have also indicated that longer operation and anesthesia time predict delirium after cardiac surgery due to increased cytokines release and operation complexity [19], which was not quite in accordance with our results.

Our results showed that patients with POD had higher CRP concentrations preoperatively and on postoperative day 2. However, the configured multivariate logistic regression model suggested plasma CRP concentrations as a predictor of POD preoperatively instead of postoperative day 2. Therefore, preoperative plasma CRP concentrations may be an important risk biomarker for POD prediction in elderly patients with colon carcinoma after laparoscopic surgery. The pathophysiologic preexisted differences in the inflammatory activity during patients before the surgery might lead to different incidences of POD. This might support our findings from a pathophysiologic standpoint and offer new important targets for investigation. However, the association between preoperative CRP concentrations and POD still remains controversial until now with different conclusions in different patient samples. Previous literature examining the relationship between POD and CRP has suggested the potential predicative role of preoperative and postoperative CRP concentrations for POD in older patients undergoing major elective surgery [20]. Other reports conducted in small samples undergoing hip and vascular surgery showed positive associations between postoperative CRP concentrations and POD [21, 22], which is not so aligned with our results. In contrast to our findings, another two studies conducted in small cohorts observed no significant association between POD and preoperative CRP concentrations [12, 23]. No close correlations were observed in critically ill medical patients [24]. Different small sample sizes of cohort-based studies, different age ranges, different surgery types, and some other confounding factors may be potential explanations for the disparate conclusions between other previous reports and our study. Those individuals with a heightened inflammatory response are at greater risk of POD occurrence as proposed by current POD pathophysiology models [25]. With no well-defined etiology of multifactorial POD, the hypothesis of inflammatory processes leading to neuroinflammation has gained wide attraction in recent years [26].

This study has some limitations. First, this study is conducted in a single-center and has a relatively small sample size in comparison with other multicenter researches. Second, the group, age range, disease diagnosis, and operation types were all relatively specific. Furthermore, the inclusion criteria of this study were not so strict and some comorbidities (such as arthritis, infections, and inflammatory diseases) might affect the results. Last, why the involved mechanisms preoperative CRP concentrations can serve as a predicator for POD still remains unclear.

In conclusion, this present study highlighted the predictive role of preoperative CRP concentrations for POD in elderly patients undergoing laparoscopic surgery for colon carcinoma. Our evidence suggested its potential role for risk stratification before the surgery from a clinical point. More intensive assessments and preventive interventions could be recommended in those patients with high risk.

## Figures and Tables

**Table 1:** Pre-, intra-, and postoperative characteristics and plasma CRP levels in patients with or without POD.

Variables	POD		*P* value
	Yes (*n* = 39)	No (*n* = 121)	
Preoperative parameters			
Age (year)	72.2 ± 5.8	69.4 ± 7.1	0.027
Gender, *n* (%)			
Male	23 (59.0%)	73 (60.3%)	
Female	16 (41.0%)	48 (39.7%)	0.881
Education, *n* (%)			
≤ high school	27 (69.2%)	71 (58.7%)	
> high school	12 (30.8%)	50 (41.3%)	0.239
BMI (kg/m^2^)	21.4 ± 3.3	22.0 ± 2.9	0.279
MCCI	1.6 ± 0.6	1.4 ± 0.5	0.041
MMSE score	25.1 ± 1.4	25.7 ± 1.6	0.038
Active smoker, *n* (%)	9 (23.1%)	30 (24.8%)	0.828
Heavy drinker, *n* (%)	10 (25.6%)	14 (11.6%)	0.032
ASA physical status, *n* (%)			
I-II	28 (71.8%)	80 (66.1%)	
III-IV	11 (28.2%)	41 (33.9%)	0.510
Intraoperative characteristics			
Operation time (min)	203.2 ± 42.1	182.6 ± 50.1	0.022
Anesthesia time (min)	239.7 ± 55.7	220.3 ± 51.1	0.045
Blood loss (ml)	230 (50–420)	220 (40–490)	0.785
Lymph node dissection			
D2	16 (41.0%)	37 (30.6%)	
D3	23 (59.0%)	84 (69.4%)	0.228
Operative approach			
Multiport surgery	28 (71.8%)	63 (52.1%)	
Single-port surgery	11 (28.2%)	58 (47.9%)	0.031
TNM classification			
I-II	24 (61.5%)	69 (57.0%)	
III-IV	15 (38.5%)	52 (43.0%)	0.619
Postoperative complications			
Incision infection	11 (28.2%)	16 (13.2%)	0.030
Urinary tract infection	4 (10.3%)	15 (12.4%)	0.719
Pulmonary infection	9 (23.1%)	12 (9.9%)	0.034
Cardiovascular events	7 (17.9%)	7 (5.9%)	0.019
Bowel obstruction	6 (15.4%)	21 (17.4%)	0.775
Anastomotic leakage	2 (5.13%)	7 (5.8%)	0.877
Postoperative bleeding	3 (7.69%)	6 (5.0%)	0.519
Plasma CRP levels (mg/L)			
Preoperatively	3.8 (0.1–38.5)	2.4 (0.1–44.2)	0.011
Postoperative day 1	48.7 ± 14.8	44.9 ± 16.2	0.196
Postoperative day 2	67.5 ± 20.1	59.7 ± 19.4	0.032
Postoperative day 3	75.1 ± 18.8	71.5 ± 21.4	0.349

POD, postoperative delirium; BMI, body mass index; MMSE, Mini-Mental State Examination; MCCI, Modified Charlson Comorbidity Index; ASA, American Society of Anesthesiologists; CRP, C-reactive protein; *P* values were calculated by Chi-square test, Fisher exact test, Mann–Whitney *U*-test, or Student's *t*-test appropriately. *P* < 0.05.

**Table 2:** Univariate and multivariate logistic regression analyses of POD in patients with colon carcinoma undergoing laparoscopic surgery.

Variables	Univariate		Multivariate	
	OR (95% CI)	*P* value	OR (95% CI)	*P* value
Age	2.87 (1.34–6.63)	0.029	1.16 (0.96–1.09)	0.46
MCCI	2.32 (1.51–3.23)	0.037	1.26 (0.49–3.12)	0.61
MMSE score	2.27 (1.14–4.67)	0.011	2.01 (0.91–4.67)	0.098
Heavy drinker	1.48 (0.89–2.53)	0.15		
Operative approach	2.34 (0.81–7.22)	0.18		
Operation time	2.12 (0.62–6.69)	0.29		
Anesthesia time	0.97 (0.92–1.04)	0.09		
Wound infection	2.83 (0.62–6.14)	0.25		
Pulmonary infection	1.37 (0.76–5.19)	0.54		
Cardiovascular events	1.28 (1.08–1.42)	<0.01	1.11 (0.87–1.68)	0.21
Plasma CRP levels				
Preoperative	11.13 (3.51–25.29)	<0.01	5.87 (2.22–11.4)	0.018
Postoperative day 2	4.54 (1.49–13.71)	0.021	3.12 (0.75–8.27)	0.34

POD, postoperative delirium; MMSE, Mini-Mental State Examination; MCCI, Modified Charlson Comorbidity Index; CRP, C-reactive protein; OR, odds ratio; CI, confidence interval. Multivariate analysis by logistic regression, *P* < 0.05.
